# Mucormycosis in a healthy elderly patient presenting as oro-antral fistula: Report of a rare incidence 

**DOI:** 10.4317/jced.52064

**Published:** 2015-04-01

**Authors:** Kumar Nilesh, Neelima A. Malik, Uzma Belgaumi

**Affiliations:** 1 M.D.S. (Oral & Maxillofacial Surgery), Reader, School of Dental Sciences, KIMSDU,Karad, India; 2 Professor & HOD, Department of Oral & Maxillofacial Surgery, School of dental Sciences, KIMSDU, Karad; 3 Lecturer, Department Of Oral Pathology, Microbiology & Forensic Odontology, School of dental Sciences, KIMSDU, Karad

## Abstract

Mucormycosis is a rare opportunistic fungal infection that commonly affects patients who are immuno-compromised. It invariably presents as an acute spreading infection, with very poor prognosis if not treated promptly. We report a case of mucormycosis in immuno-competent elderly patient, presenting as oro-antral communication. Patient’s history, clinical and laboratory evaluation revealed no systemic predisposing factors. The disease was non-fulminant, localized and showed remission after local measures, without parentral anti-fungal therapy.

** Key words:**Mucormycosis, maxilla, elderly, oroantral communication.

## Introduction

Mucormycosis, also known as zygomycosis, is a rare, life-threatening fungal infection caused by saprophytic organism of the order mucorales (1). Most of the infection is caused by genera *rhizopus*, *mucor*, and *rhizomucor*. These fungi usually do not cause disease in healthy people with intact immune systems. However a number of conditions can predispose to development of this disease, including diabetic ketoacidosis, renal failure, malignancies, intravenous drug abuse, malnutrition, neutropenia due to haematological disease (leukaemia, lymphoma or aplastic anaemia), anti-neoplastic drugs, organ transplant, acquired immune-deficiency syndrome and chronic corticosteroid therapy (2). If not recognized early or treated inadequately, they are among the most acutely fatal infections known.

This paper reports a rare case of mucormycosis in a healthy elderly patient, presenting as a chronic oroantral communication. The patient’s history revealed no predisposing factors, nor the clinical and haematological evaluation gave any indication of mucormycosis, thus presenting as a diagnostic dilemma. Unlike acute and fulminant nature of mucormycosis, the disease was self-limiting and showed remission after local measures without parentral anti-fungal therapy. The protocol of the present report has been approved by the ethical review board of the author’s institution.

## Case Report

A 72 years old male patient and farmer by occupation presented with complaint of escape of fluid from his left nostril after drinking water since one month. There was no associated pain, swelling or discomfort. Patient gave history of traumatic extraction one month earlier at rural dental hospital. No significant medical or family history was reported. Extra-oral examination showed no apparent abnormality. On intra-oral examination, the patient had a partially edentulous maxillary arch with non-healing extraction sockets of maxillary left molars. Dehiscence of gingival tissue and exposed alveolar crestal bone was noted at the site of extraction. Escape of water through left nostril was demonstrated on oral intake. Panoramic radiograph of the jaw bone showed a linear radiolucency extending from alveolar crest to floor of maxillary sinus, with breach in continuity of the sinus floor, suggestive of oro-antral communication (OAC). Routine blood examination, including serum glucose and haemogram of the patient was within normal limits. Diagnosis of OAC subsequent to extraction of maxillary molar teeth was established. Patient was put on pre-operative antibiotics (Tablet Amocicillin 500mg + Potassium Clavulanate 125 two times a day) and nasal decongestant. Surgical closure of the OAC was planned and executed under local anaesthesia.

**Figure 1:**
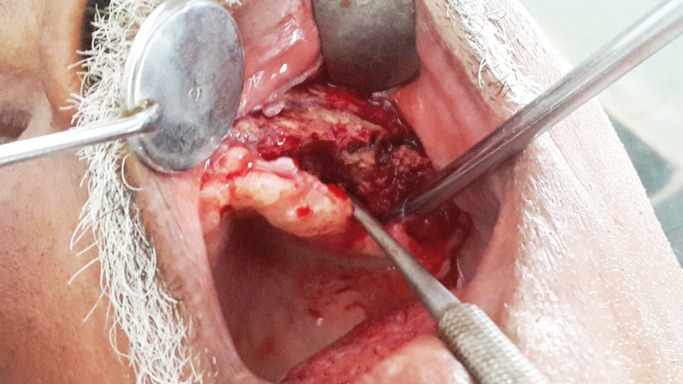
Intra-operative views: Necrotic alveolar bone extending from midline to left maxillary tuberosity region.

**Figure 2:**
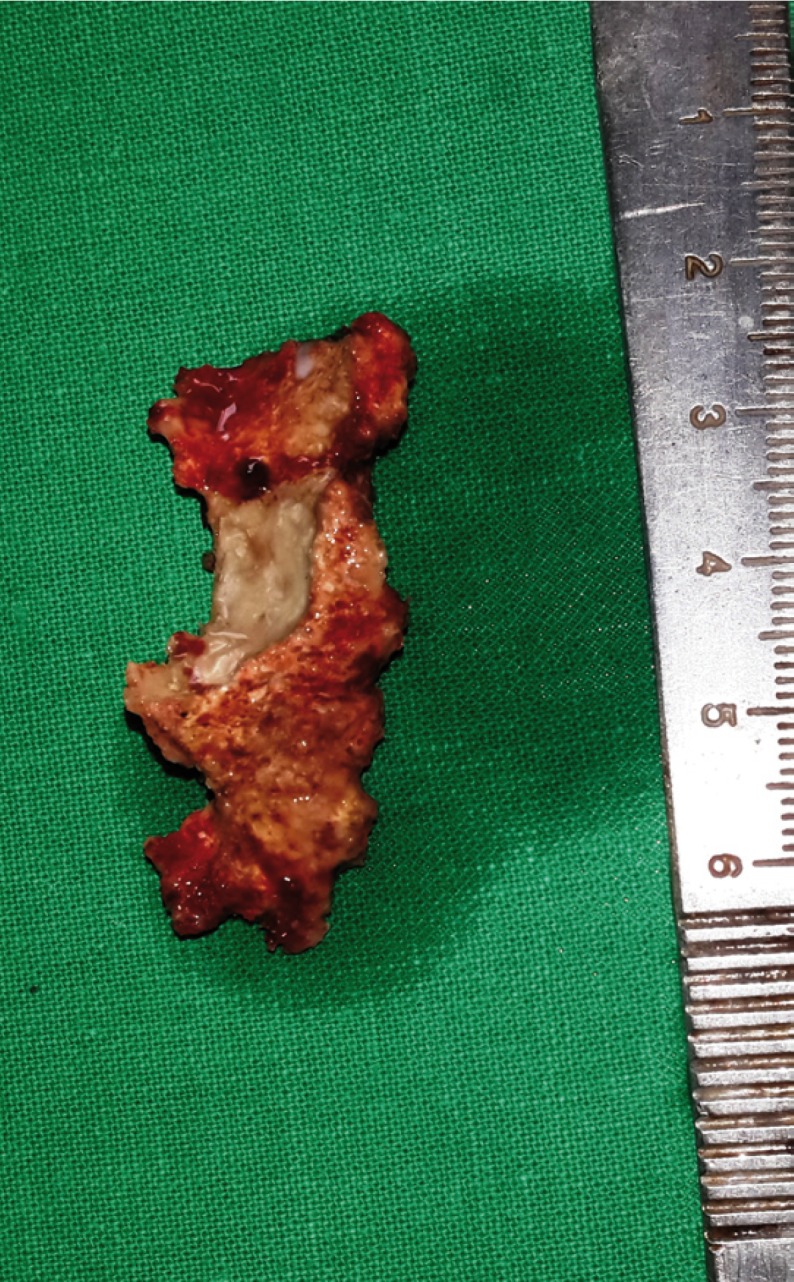
Intra-operative views: excised alveolar bone.

**Figure 3:**
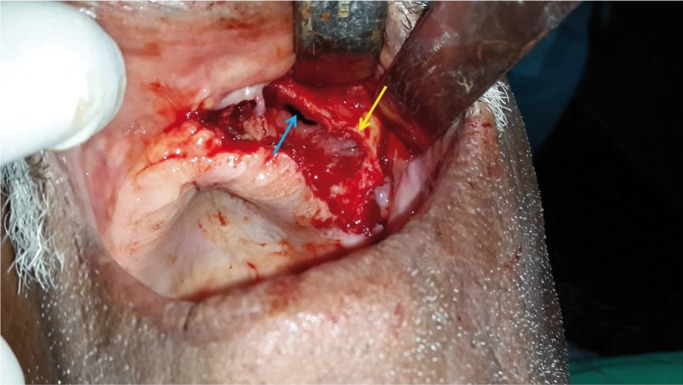
Intra-operative views: Surgical site after removal of the alveolar bone. Note the healthy bone at maxillary buttress region (white arrow) and breach in sinus lining (black arrow).

Intra-operatively on reflection of buccal and palatal mucosal flaps, necrotic maxillary alveolar bone was seen extending from midline to left maxillary tuberosity region, including the extraction sockets of molars (Fig. 1). The block of maxillary left alveolus was mobile and easily elevated from its soft tissue attachments (Fig. 2). Interestingly the bone superior to the alveolus, in maxi-llary buttress region appeared completely healthy (Fig. 3). The area was irrigated and after attaining adequate haemostasis the defect was closed primarily, by advancing the buccal flap and suturing it to the palatal mucosa. The necrotic maxillary alveolar bone was submitted for histopathologic evaluation. Computed Tomography (CT) was advised postoperatively, to study extent of skeletal involvement. The CT scan showed thickening of the left maxillary sinus lining.

**Figure 4:**
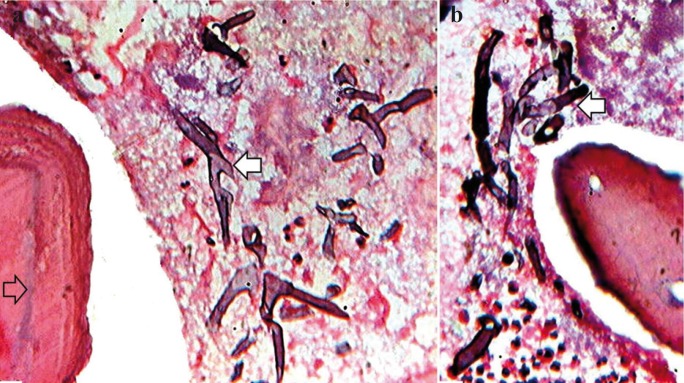
Photomicrograph showing a) presence fungal hyphae (block arrow), bone trabaculae (hollow arrow) and b) broad aseptate hyphae with acute irregular branching pattern characteristic of mucormycosis (hematoxylin and eosin stained, ×400).

Microscopic study of the alveolar bone revealed presence of broad aseptate hyphae with acute angle branching pattern (Fig. 4a) surrounded by area of bone trabaculae (Fig. 4b). Based on the histopathologic examination diagnosis of mucormycosis was established.

The patient was subjected to through clinical and laboratory examination, including total and differential blood lymphocyte count, peripheral blood smear, liver function test, fasting and postpranadial blood sugar levels, serum immunoglobulin levels, liver function tests (LFT), to rule out underlying systemic and immunosuppressive diseases. Considering the fulminant and fatal course of the fungal infection, hospitalization and parenteral antifungal therapy was advised. However the patient was not willing for admission. Oral antifungal therapy (Posaconazole; 400 mg twice daily for 6 weeks) was prescribed and the patient was kept on daily recall. The post-operative healing was uneventful and the infection showed complete remission. At 6- month follow up there was satisfactory healing of the surgical site. On radiological assessment no significant finding was seen and both the maxillary sinuses appeared normal.

## Discussion

Mucormycosis is a rare fungal infection, first reported in humans by Paultaufin in 1885 (3). It is also called as zygomycosis and is caused by fungi belonging to the order Mucorales. Most of the species known to cause human disease belong to the family Mucoraceae, which includes the *Rhizopus*, *Mucor*, and *Absidia* genera. These fungi exist in environment in soil, air, food, composite piles, and animal excreta and play a major role in decomposition in the natural world. The major mode of disease transmission for the fungi is presumed to be via inhalation of spores from environmental sources. Less often, primary mucormycosis is caused by implantation of fungal elements in traumatic cutaneous lesions, burns or in association with intravenous drug abuse. Our patient was a farmer by occupation and would have possibly got infected with the fungi by inhalation of sporangiospores from the atmosphere or contact with contaminated soil.

Mucormycosis is characterized by rapid growth rate and typically cause acute, aggressive, and frequently angioinvasive infec-tions, especially in immunosuppressed hosts. Its clinical presentation is variable and depends upon the site of entry of micro-organism and organ systems involved. The most common form is rhinocerebral, involving the nose, paranasal sinuses, orbits and central nervous system. Other forms of mucormycosis include cutaneous, gastrointestinal, pulmonary and disseminated. Early symptoms of rhino-cerebral mucormycosis are peri-nasal paresthesia, cellulitis, periorbital edema, rhinorrhea and nasal crusting. These features are quickly superseded by eschar formation and necrosis of the naso-facial region (4). Unlike the acute and fulminant course of this infection, our case presented as a localized necrosis of left maxillary alveolar bone, in an immune-competent patient. The only clinical finding on presentation was an OAC subsequent to extraction of maxillary molar teeth.

Mucormycosis typically colonizes and widely spreads in susceptible immune-compromised host by vascular invasion with subsequent tissue necrosis and infarction. The possible hypothesis to explanation the localized nature of the infection in our patient can be the compromised local host defense mechanism. Mucosal epithelium and vascular endothelium presents an effective barrier against tissue invasion by micro-organisms. Epithelium and endothelium damaged by prior infection (chronic sinusitis in our case, as evident on post-operative CT scan) and trauma (traumatic tooth removal) could have made this otherwise systemically healthy individual, prone to acquire the infection.

Despite many advances in diagnosis and treatment, mucormycosis still carries a high mortality rate (75–95%) (1). Management of mucormycosis requires hospitalization and combination of parenteral antifungal therapy, surgical intervention, and control of the underlying risk factors. Unlike the acute and fulminant course of the disease, our case presented as a self-limiting, localized low-grade fungal infection in an immuno-competent hosts. Also to best of our knowledge this is the first case report of oral mu-cormycosis in english literature, presenting as OAC.
